# Opposite Effective Connectivity in the Posterior Cingulate and Medial Prefrontal Cortex between First-Episode Schizophrenic Patients with Suicide Risk and Healthy Controls

**DOI:** 10.1371/journal.pone.0063477

**Published:** 2013-05-21

**Authors:** Huiran Zhang, Xiaomei Wei, Haojuan Tao, Tumbwene E. Mwansisya, Weidan Pu, Zhong He, Aimin Hu, Lin Xu, Zhening Liu, Baoci Shan, Zhimin Xue

**Affiliations:** 1 Mental Health Institute of The Second Xiangya Hospital, Central South University, Changsha, China; 2 Key Laboratory of Nuclear Analysis Techniques, Institute of High Energy Physics, Chinese Academy of Sciences, Beijing, China; 3 Department of Psychiatry, The First Affiliated Hospital of Zhengzhou University, Zhengzhou, China; 4 Department of Clinical Nursing and Community Health, the University of Dodoma, Dodoma, Tanzania; 5 Department of Radiology of The Second Xiangya Hospital, Central South University, Changsha, China; 6 Key Laboratory of Animal Models and Human Disease Mechanisms, Kunming Institute of Zoology, Chinese Academy of Sciences, Kunming, China; University of Medicine & Dentistry of NJ - New Jersey Medical School, United States of America

## Abstract

**Objective:**

The schizophrenic patients with high suicide risk are characterized by depression, better cognitive function, and prominent positive symptoms. However, the neurobiological basis of suicide attempts in schizophrenia is not clear. The suicide in schizophrenia is implicated in the defects in emotional process and decision-making, which are associated with prefrontal-cingulate circuit. In order to explore the possible neurobiological basis of suicide in schizophrenia, we investigated the correlation of prefrontal-cingulate circuit with suicide risk in schizophrenia via dynamic casual modelling.

**Method:**

Participants were 33 first-episode schizophrenic patients comprising of a high suicide risk group (N = 14) and a low suicide risk group (N = 19). A comparison group of healthy controls (N = 15) were matched for age, gender and education. N-back tasking functional magnetic resonance imaging data was collected.

**Results:**

Compared with healthy controls group, the two patients groups showed decreased task-related suppression during 2-back task state versus baseline state in the left posterior cingulate and medial prefrontal cortex; the hyper-connectivity from the left posterior cingulate cortex to the left medial prefrontal cortex existed in both schizophrenic patients groups, but hypo-connectivity in the opposite direction only existed in the schizophrenic patients group with high suicide risk.

**Conclusions:**

The hyper-connectivity from the left posterior cingulate cortex to the left medial prefrontal cortex may suggest that the abnormal effective connectivity was associated with risk for schizophrenia. The hypo-connectivity in the opposite direction may represent a possible correlate of increased vulnerability to suicide attempt.

## Introduction

It is well known that schizophrenia contributes substantially to the global burden of disease and is commonly associated with suicide [1]. Dutta et al reported that suicide in first episode psychosis occurs approximately 12 times more than the general population [2]. In patients with schizophrenia, the rate of suicidal attempts can be almost up to 40% higher [3]. Some studies suggested that the schizophrenia patients with high suicide risk are characterized by depression, better cognitive function, prominent positive symptoms and low negative symptoms [4]–[7]. However, the neurobiological basis of suicide attempts in schizophrenia is not clear. The previous studies considered that suicidal behavior was associated with dysfunction of decision-making, problem solving and emotional process [8].

Functional neuroimaging studies have found that the posterior cingulate cortex (PCC) and the medial prefrontal cortex (MPFC) mediate the performance of decision making, social cognitive tasks, and emotional process [9], [10]. At the same time, the pathology of the prefrontal cortex and limbic system existed in schizophrenia. The MPFC, in particular, is sensitive to the emotional valence of information [11] and implicated in the processing of personal preferences, judgments about the subjective value of future outcomes and the pursuit of self-specified goals [12]–[14]. The PCC is implicated in episodic memories and emotional process [15]. Goal setting and planning is likely to base on the MPFC-PCC circuit that has shown to regulate self-initiated goal formations about personally desired future events [16]. Hence the abnormal activities and connectivities in the MPFC and PCC may lead to the disorganized goal setting and planning, improper estimation of future outcomes, assignment of self-reference to unrelated external events. On this basis, we guess that the impaired MPFC-PCC activities and connectivities may be associated with the high suicide risk in schizophrenia.

Relevant evidence between vulnerability to suicidal behavior in schizophrenia and neurobiological abnormalities is accumulating, but imaging studies about suicide behavior in schizophrenia are few. While previous studies mainly focus on the lesion of local brain regions (such as fronto-limbic brain structures), studies on abnormal information transmission in neural circuits contributing to suicide behavior in schizophrenia are scarcely available [17]–[19]. Tracts between the MPFC and PCC have been demonstrated in macaques [20]. Greicius and his colleagues have demonstrated that within the default mode network (DMN) tracts from the MPFC enter into the PCC, suggesting the MPFC have direct projections to the PCC [21], such that, we can infer that there is a flow of information transmission between the MPFC and the PCC. Previous studies investigated the coupling between MPFC and PCC in terms of temporal correlations, that is “functional connectivity” [22], and could not indicate the direction of the impaired connectivity. In view of the shortcoming of temporal correlations, we used the dynamic causal modeling (DCM) analysis to characterize the aberrant effective connectivity between MPFC and PCC in schizophrenia individuals with suicide risk.

DCM which can show the direction of the connectivity is used to estimate and make inferences about coupling in various brain areas; also DCM can show how such coupling is influenced by changes in the experimental context [23], [24]. This procedure sheds light on how one neural system exerts influence over another, and how it can be affected by the experimental context. We used the DCM which previously was validated by biophysical model of functional magnetic resonance imaging (fMRI) measurements to predict the underlying neuronal activity from the observed hemodynamic response. We hypothesized that the special aberrant effective connectivity would be associated with suicide risk in schizophrenia. Such a pilot study would provide insight into the neurobiological basis of suicidal behavior.

## Methods

### Participants

Using the Structured Clinical Interview for DSM-IV patient version (SCID-P) [25], thirty three first-episode schizophrenic patients were recruited from inpatient and outpatient units of the Department of Psychiatry, the Second Xiangya Hospital of Central South University, Changsha, Hunan province, PR China. The inclusion criteria were: a) age between 18 and 45; b) Han Chinese ethnicity; c) nine years of education or above; d) right-handed by a determination of hand preference; e) met DSM-IV criteria for schizophrenia; f) the total course of disease≤18 months. The exclusion criteria were: a) any contra-indications to MRI scanning; b) substance-related disorders; c) disorder of consciousness of more than five minutes; d) chronic neurological disorders or severe physical diseases. Risk of suicide was quantified using the Schizophrenia Suicide Risk Scale (SSRS) [26]. Patients were divided into the following two groups: high suicide risk group (HSR) (n = 14) with SSRS score≥17; low suicide risk group (LSR) (n = 19) with SSRS score<17.

Fifteen healthy controls (HC) were recruited from a community sample in Changsha city. The inclusion and exclusion criteria were the same as those of the patients group except that the healthy controls did not meet the DSM-IV diagnostic criteria of any psychiatric disorders by SCID non-patient version.

All participants gave written informed consent for participation in the study after the risks and benefits of their participation were explained in detail. The study was approved by the ethics committee of the Second Xiangya Hospital of Central South University.

### N-back Task

Participants were presented with a letter n-back task with two experimental conditions: 0-back, and 2-back [27]. The n-back task was performed on NordicNeurolab’s fMRI hardware system, on a computer screen at 2 s intervals in 40 s blocks. In the 0-back condition, participants were required to press the right button on the response pad when a single pre-specified target letter (e.g., “X” or “x”) appeared. In the 2-back condition, they were required to press a button on the response pad with their right index finger if the letter currently visible was identical to the one presented two trials back. The stimuli were pseudorandom sequences of letters. The experiment utilized a blocked design with four epochs for each of the two experimental conditions (8 epochs total) with 20 letters per epoch and targets occurring seven times in each epoch. At the beginning of each epoch, a visual instruction indicated the condition (0-back or 2-back) for 2 s. Each stimulus letter was presented for 500 ms, followed by 1500-ms delay. A fixation epoch, in which one crossing for fixation (+) was presented for 20 s, was inserted after every epoch, resulting in a total of eight fixation epochs. The data from these fixation epochs was used as the baseline condition. At the beginning of the session, a short fixation epoch presented for 4 s was inserted to allow the magnetization to reach equilibrium amplitude. The functional task-state session lasted 8 minutes and 20 seconds. The reaction time and the accuracy of the responses were recorded on-line.

### Image Acquisition

Scans were performed on a Philips Gyroscan Achieva 3.0 Tesla MRI scanner. Functional magnetic resonance imaging (fMRI) images were collected in the axial location, using a gradient-echo echo-planar imaging (EPI) sequence: repetition time (TR) = 2000 ms, echo time (TE) = 30 ms, flip angle (FA) = 90°, matrix = 64×64, slice thickness = 4 mm, gap = 0 mm, slices = 36. Each functional task-state session lasted 8 minutes and 20 seconds, 250 volumes were obtained.

### Functional MRI Image Preprocessing and Statistical Analysis

All functional images were processed on Statistical Parametric Mapping 8 (SPM8; http//www.fil.ion.ucl.ac.uk/spm ). For each participant, the first two volumes of scanned data were discarded because of the magnetic saturation effects. The following preprocessing was applied to the remaining 248 volumes of each subject: slice timing was used to correct differences in slice acquisition times. Temporal processed images were realigned to the first volume for correcting head motion and a mean functional image was correspondingly obtained. The participants must have matched up to the criterion that less than 1 mm of translation and 1° of rotation in x, y, or z axis. The realigned images from each participant were normalized to the EPI template to fit into a standard anatomical space. The normalized voxel size was oversampled to 3×3×3 mm. All normalized images were then smoothed with an 8 mm Gaussian kernel.

The first level analysis (subject-specific analysis) was performed to identify regional activities for all brain voxels in each participant, and contrasts were created for each participant comparing baseline condition versus 2-back task condition. The subject-specific contrast images were then entered into a second level ANOVA analysis to make inferences at the group level. We found two regions that were consistently and differentially activated in the different groups. These regions were in the left MPFC and PCC. These two areas were then entered into our DCM analysis in order to test the abnormal effective connectivity between the MPFC and PCC.

### DCM Analysis

Our DCM analysis aimed to find differences in prefrontal-cingulate coupling among the three groups. We addressed these potential differences at two levels. First, we used Bayesian model comparison to assess the best model for each group. This allowed us to assess the contribution of different connections, in terms of whether they were present or absent under different models. We then assessed group differences in connection strengths quantitatively using classical statistics (ANOVA) based upon the connection strengths estimated for each subject. To provide an estimate of each connection, for each subject, we used Bayesian model averaging (as implemented in SPM) to account for our uncertainty about which model was the best explanation for the data from each subject. Put simply, Bayesian model averaging weights the estimates for each model in proportion to the evidence for different models. We constructed five dynamic causal models for each subject. Each model included two areas: the left MPFC and PCC. The left MPFC [mean coordinates (x, y, z): –3, 56, 22] and the PCC [mean coordinates (x, y, z): 0, −49, 28] were selected on the basis that they showed significantly differential activations across the three groups during baseline state relative to 2-back task in the standard SPM analysis. Principal Eigen variates were extracted to summarize regional responses in 6 mm spheres centered on the regions included in the DCM. To account for individual differences, the location of these regions was based upon the local maxima of the subject-specific statistical parametric maps, defined as the nearest maxima (within 6 mm) of the group maxima.

## Results

### The Description of Demographic and Clinical Characteristics

Demographic and clinical characteristics for the three groups were summarized in Table 1. There were no significant differences in age, gender, education and the response time for correct responses during 2-back task among the three groups (all *p*>0.05). There were significant differences in scores of the Information (I) subscale (*F = *4.487, *p = *0.017) and the Digit Symbol (DS) subscale (*F = *19.442, *p<*0.001) of the Wechsler Adult Intelligence Scale-Chinese Revised (WAIS-CR), as well as the proportion of correct responses during 2-back task (*χ^2^* = 64.245, *p<*0.001). In the two patients groups, there were no significant differences in course of disease, duration of treatment and dosage of chlorpromazine equivalents (all *p*>0.05). There were significant differences in the scores of the Scale for the Assessment of Positive Symptoms (SAPS) (*t = *2.635, *p = *0.013) [28], the scores of the Scale for the Assessment of Negative Symptoms (SANS) (*t* = −3.426, *p = *0.002) [29] and the scores of SSRS (*t = *6.639, *p<*0.001).

**Table 1:** The demographic and clinical characteristics for high suicide risk group (HSR), low suicide risk group (LSR) and healthy controls group (HC).

Variable	HSR	LSR	HC	Group comparisons
Age (year)	22.86 (4.00)	24.47 (6.78)	24.13 (6.37)	*F* = 0.313, *p = *0.733
Education (years)	12.18 (2.30)	12.08 (2.24)	13.17 (2.36)	*F* = 1.076, *p = *0.349
Gender (male/female)	8/6	10/9	6/9	*χ^2^* = 0.938, *p = *0.626
WAIS-I score	16.07 (4.64)	15.76 (3.54)	19.70 (4.12)	*F = *4.487, *p = *0.017*
WAIS-DS score	67.14 (16.71)	63.05 (11.52)	91.40 (13.60)	*F = *19.442, *p<*0.001*
SAPS scores	22.21 (9.42)	13.21 (9.90)	–	*t = *2.635, *p = *0.013*
SANS scores	18.57 (10.03)	41.95 (27.35)	–	*t* = −3.426, *p = *0.002*
SSRS scores	20.93 (5.51)	10.68 (3.33)	–	*t = *6.639, *p<*0.001*
Course of disease (months)	6.93 (3.95)	9.91 (5.62)	–	*t* = −1.697, *p = *0.100
Duration of treatment (days)	14.21(10.23)	9.21 (9.01)	–	*t = *1.489, *p = *0.147
CPZ equivalents	254.76 (192.09)	325.88 (285.19)	–	*t* = −0.806, *p = *0.426
Proportion correct responses	0.52	0.59	0.78	*χ^2^* = 64.245, *p<*0.001*
Response time (s) for correct responses	0.73 (0.14)	0.71 (0.19)	0.63 (0.11)	*F = *1.573, *p = *0.219

Data reflect mean (SD) unless otherwise stated. “–” reflect not applicable; “*” means significant statistical difference (statistical threshold *p*<0.05). WAIS-I = Information subscale of the Wechsler Adult Intelligence Scale-Chinese Revised; WAIS-DS = Digit Symbol subscale of the Wechsler Adult Intelligence Scale-Chinese Revised; SAPS = Scale for the Assessment of Positive Symptoms; SANS = Scale for the Assessment of Negative Symptoms; SSRS = Schizophrenia Suicide Risk Scale; CPZ = Chlorpromazine. The proportion correct responses and response time(s) for correct responses were the performance during 2-back task.

### fMRI

**Figure 1:**
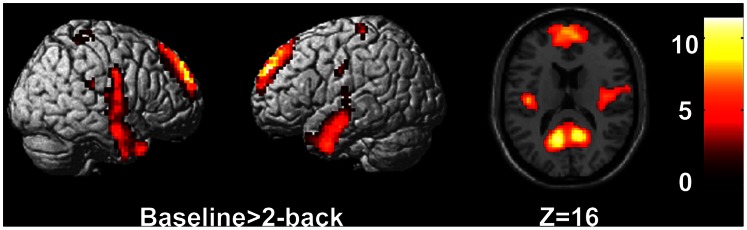
Regions with significant activations at rest state compared with 2-back task (*p*<0.05, FWE corrected).

**Figure 2:**
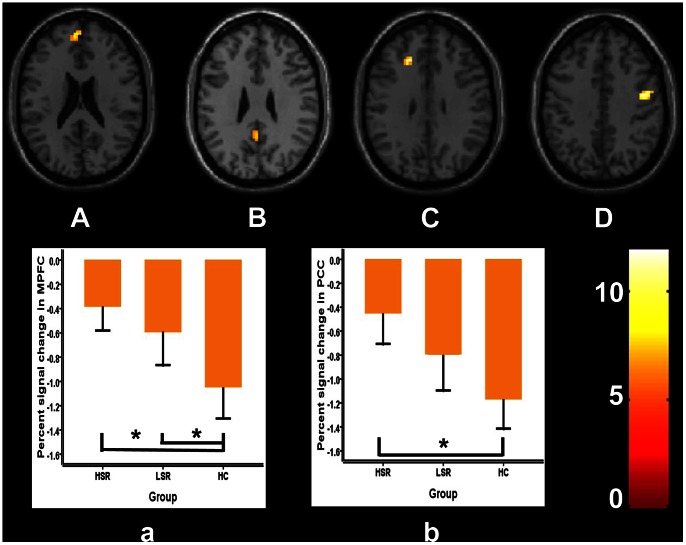
Regions with significant activations at rest state among three groups compared with 2-back task. From A to D (upper part), brain areas are left medial prefrontal cortex (MPFC), left posterior cingulate cortex (PCC), left middle frontal gyrus and right precentral gyrus (*p*<0.005, cluster >20). The signal changes in MPFC and PCC in the three groups are shown below (a and b). Error bars indicated 2 standard errors (SE), *p<0.05.

The subject-specific analysis was firstly performed to identify the activated brain regions in each participant on the baseline condition versus 2-back condition. The subject-specific results were then entered into a second level analysis to obtain the activated areas in each group using one-sample t-test. In this step, we needed to correct for multiple comparisons over the entire brain to identify the brain activation, such that we need to set up a quite strict statistical threshold at p<0.05, FWE corrected, cluster >100 to control the Type I (false positive) error. The results have shown that the brain activity of the posterior cingulate cortex (PCC), the medial prefrontal cortex (MPFC) and middle temporal gyrus were significantly suppressed during 2-back task in all three groups (one-sample t-test, *p*<0.05, FWE corrected, cluster>100; Figure 1). We then used ANOVA analysis to assess task-specific regional suppression across the HSR, LSR and HC groups. In this step, because group differences constitute a group by condition interaction, they are orthogonal to the main effect of the task induced suppressions. This means that we did not need to correct for multiple comparisons over the entire brain. Therefore, we restricted our search for group differences to the regions showing a main effect of task by using an uncorrected p-value of 0.005 and cluster >20. This p value also has been chosen by other authors [30]–[34]. The significantly differential activations during baseline versus 2-back task among the three groups were the left posterior cingulate cortex (PCC), the left medial prefrontal cortex (MPFC), the left middle frontal and the right precentral gyrus (ANOVA, *p*<0.005, uncorrected, cluster >20; Figure 2). Furthermore, in order to confirm the task-specific suppression regions, additional Small Volume Correction (SVC) has been performed (using an 8 mm radius sphere centered on the coordinates in MPFC [mean coordinates (x, y, z): –3, 56, 22] and in PCC [mean coordinates (x, y, z): 0, −49, 28]) [34]. We found that task-specific suppression in the MPFC and PCC remain significant after SVC (*p*<0.05, FWE correction). In the left PCC, post hoc tests indicated that the brain activity in HC group was lower than that in HSR group (*p = *0.001), without significant difference between other groups (*p = *0.057 for HC vs. LSR and *p = *0.090 for HSR vs. LSR). In the left MPFC, post hoc tests indicated that the brain activity in HC group was lower than that in both patients groups (*p = *0.001 for HC vs. HSR and *p = *0.014 for HC vs. LSR), without significant difference between HSR and LSR (*p = *0.248). Interestingly, task-related suppression of MPFC and PCC showed a sequentially decreased trend from HSR to LSR to HC (Figure 2). We also found that the activity of MPFC was positively correlated with the scores of SSRS (*r* = 0.451, *p = *0.027).

### Dynamic Causal Modelling

**Figure 3:**
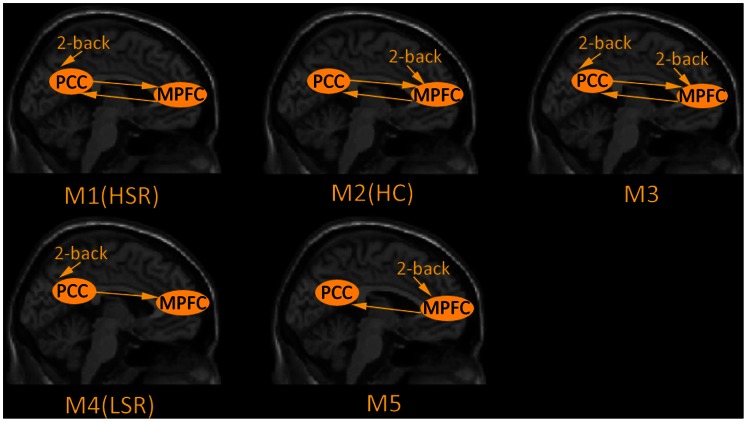
Five hypothesized dynamic casual models during 2-back task. Each model includes the left medial prefrontal cortex (MPFC) and the ipsilateral posterior cingulate cortex (PCC), in which the arrows indicate extrinsic stimulation and functional connections direction. M1, M2 and M3 comprised bi-directional connections with respectively driving input into PCC or MPFC or both of the regions. M4 only contained unidirectional connection from the PCC to MPFC with driving input into PCC and M5 was completely opposite to M4. M1, M4 and M2 were identified as the optimal models in HSR, LSR and HC respectively. HSR: schizophrenic patients with high suicide risk; LSR: schizophrenic patients with low suicide risk; HC: healthy controls.

We considered five models that are shown in Figure 3. These five models involved either bi-directional or uni-directional connections between the MPFC and PCC and allowed for the extrinsic effect of the 2-back task to enter into one or other or both of the regions. In detail, Model 1 (M1), Model 2 (M2) and Model 3 (M3) comprised bi-directional connections with driving respectively input into PCC or MPFC or both of the regions. Model 4 (M4) only contained unidirectional connection from the PCC to MPFC with driving input into PCC and Model 5 (M5) was completely opposite to M4. We used Bayesian model selection (BMS) to optimize the DCM models and found that the optimal models were M1 in HSR group, M4 in LSR group and M2 in HC group.

We were interested in the strength of the endogenous connections which refer to the impact that one region exerts over another during task performance. To provide an estimate of each endogenous connection, for each subject, we used Bayesian model averaging to account for our uncertainty about which model was the best explanation for the data from each subject. The endogenous connections in three groups are shown in Table 2. When all participants were combined, the endogenous connections were significantly greater than zero, indicating strong functional integration during task performance. Group comparisons revealed that the endogenous connection from MPFC to PCC was significantly different among three groups (ANOVA; *F* = 156.158, *p*<0.001). Post hoc, two-tailed t-tests showed that this connection was stronger in HC group than HSR group (*p*<0.001) and LSR group (*p*<0.001), but did not differ between the two patients groups (*p = *0.155). The endogenous connection from PCC to MPFC across three groups was also significantly different (ANOVA; *F* = 24.210, *p*<0.001). Post hoc, two-tailed t-tests showed that this connection was stronger in both HSR group (*p*<0.001) and LSR (*p*<0.001) group than HC group, but did not differ between the two patients groups (*p = *0.793).

**Table 2:** Endogenous connections weighted by Bayesian model average in high suicide risk group (HSR), low suicide risk group (LSR) and healthy controls group (HC).

Endogenous connections	HSR	LSR	HC	All groupscombined (*p*-values)	GroupComparisons (*p*-values)
				Endogenous connections	Endogenous connections
MPFC→PCC	0.13(0.10)	0.06(0.05)	0.78(0.17)	<0.001*	<0.001*
PCC→MPFC	0.62(0.13)	0.64(0.15)	0.28(0.15)	<0.001*	<0.001*

Group mean estimates and SD (in brackets) are reported for endogenous connections which was weighted by Bayesian model average, the endogenous connections of all groups combined means that were significantly greater than zero across the three groups. We also report the group differences, which were identified using a series of ANOVAs. PCC = posterior cingulate cortex; MPFC = medial prefrontal cortex; “*” means significant statistical difference (statistical threshold *p*<0.05).

In summary, both the qualitative model comparison and quantitative comparison of parameters weighted by Bayesian model average (in relation to inter-subject variability) painted the same picture: namely a loss of or reduction in effective connectivity from the medial prefrontal cortex to the posterior cingulate cortex in schizophrenics at lower risks of suicide - relative to those at high risk.

## Discussion

The aim of this study was to examine the possible neurobiological basis in schizophrenic patients with high suicide risk using n-back task fMRI data via DCM analysis. In the current study, patients with schizophrenia and healthy controls demonstrated opposite patterns of effective connectivity between MPFC and PCC. First, in these two cortical midline regions, the two patient groups (LSR and HSR) showed hyperactivity at 2-back task state compared with baseline. Second, the LSR group was unidirectional hyper-connectivity from PCC to MPFC. HSR and HC were bidirectional effective connectivity between PCC and MPFC. Compared with healthy controls, schizophrenic patients with high suicide risk showed hyper-connectivity from PCC to MPFC, and hypo-connectivity in the opposite direction.

The MPFC and PCC are directly involved in decision making, goal-directed behavior and emotional processing [35], [36]. The concept of motivational salience contains the decision making, which can explain and understand the evaluations and choices that humans make [37]. In schizophrenia, the general hypothesis was that motivational salience is mis-assigned to unimportant, neutral or affectively ambiguous information [38]. In this study, we found that reduced suppression occurred in both patient groups during 2-back task. Hyper-activation of the MPFC and PCC may constantly divide or misdirect the attention resources when patients attempt to perceive, think, or act on the external environment. When the brain does not properly process social information, it would become difficult to perform adaptive motivated behaviors in response to the information, in both present and future contexts. Normal brains need to form learned associations between social information cues in our environments and their associated emotional meanings, as well as perform adaptive emotional context [39]. For the schizophrenic patients, it is possible that the emotional valence of social information and the subjective value of future outcomes are inappropriately estimated, finally leading to their biased decisions.

The previous study found that MPFC dysfunction was associated with risk for schizophrenia, whereas PCC dysfunction was associated either with the greater risk for the disease or the expression of the illness [40]. The other study has demonstrated that the MPFC have direct fiber projections to the PCC [21]. We speculate that the motivational and emotional information was integrated in MPFC, and then the information fed back to PCC to modulate the motivation and emotion at a relatively stable state and prompted the external expression of motivation and emotion. We found that the activity of MPFC was positively correlated with the scores of SSRS and the hypo-connectivity from MPFC to PCC only existed in HSR among two patients groups. We speculate that maybe the hypo-connectivity from MPFC to PCC is correlated with the high suicide risk in schizophrenia. In LSR group, the PCC could not receive information from the MPFC due to the absence of feedback, which may lead to the malfunction of motivational behavior and emotion expression. Compared with healthy controls, the hypo-connectivity from MPFC to PCC may result in a weakened regulation from MPFC to PCC in HSR group. Hyper-connectivity from PCC to MPFC was a prominent difference between patients with schizophrenia and healthy controls. More improperly processed social information from PCC was transmitted to MPFC, may lead to the confusion goal setting and planning, improper estimation of future outcomes, and assignment of self-reference to unrelated external events.

The previous study found that the prominent negative symptoms may prevent the emergence of suicide in patients with schizophrenia and the prominent positive symptoms (in particularly the suspiciousness and delusions) may increase the suicide risk [4]. In this study, compared with the schizophrenic patients with low suicide risk, the schizophrenic patients with high suicide risk were characterized by prominent positive symptoms, lower levels of negative symptoms. Some studies suggests that the patients with schizophrenia spectrum disorders who have suicide behavior had a better cognitive function than those who have not [7]. Interestingly, in the present study, we did not found a significant difference pertaining to the WAIS scores between HSR and LSR. One possibility is that we only chose the Information (I) subscale and the Digit Symbol (DS) subscale of the Wechsler Adult Intelligence Scale-Chinese Revised (WAIS-CR) to assess the intelligence, which possibly could not reflect the overall level of the intelligence. Future work devoted to assessing overall intelligence differences between HSR and LSR is warranted. It is widely believed that schizophrenia is characterized by longer reaction time and less accurate working memory performance. Whereas some studies did not show significant difference in reaction time between schizophrenic patients and healthy controls [41], [42]. In our study, compared with healthy controls, the schizophrenic patients showed a trend to have longer response time (Table 1). These inconsistent findings may be a consequence of insufficient statistic power owing to our relatively small sample, and larger samples will be required to evaluate them.

Our result is consistent with previous reports of functional integration impairment in schizophrenia and, in particular, adds to the evidence for a specific disturbance of the functional coupling between prefrontal and cingulate regions. Our data further extend the previous findings with respect to two main aspects. First, this is a pilot study on the possible neurobiological basis of schizophrenia patients with high suicide risk using n-back task fMRI data via DCM analysis. The previous studies investigated the coupling between medial prefrontal cortex and cingulate formation in terms of temporal correlations, that is “functional connectivity” [22], and could not indicate the direction of the impaired connection. By using dynamic casual modeling [23], we were able to show the altered connectivity between the cingulate formation and medial prefrontal cortex. Second, we investigated the interaction between cingulate and medial prefrontal cortex function in patients who had just developed schizophrenia as we did not use chronic patients. This minimized the risk that the findings were secondary to an effect of chronic illness or its treatments. Both functional and structural abnormalities in schizophrenia may change over the course of psychotic disorders [43] and antipsychotic medication can modify activation during cognitive tasks [44], [45].

### Conclusion

In summary, using fMRI and dynamic casual modeling, we found that the hyper-connectivity from PCC to MPFC existed in the two patients groups, relative to healthy controls. This suggests that the abnormal effective connectivity is probably associated with risk for schizophrenia. Compared to healthy controls, the hypo-connectivity from MPFC to PCC only existed in HSR group. This abnormality may indicate a possibility of increased vulnerability to suicide attempt.
